# Supplementary material for: Are carbon emissions trading and green financial instruments synergistic? -Comprehensive quantitative research based on content analysis
Source: PLoS One. 2024 Mar 7;19(3):e0298601. doi: 10.1371/journal.pone.0298601 (PMC10919601; doi:10.1371/journal.pone.0298601)
Supplement: S1 Data — (ZIP) [file pone.0298601.s001.zip › Data/地级市Z_F工作报告.zip!/║┌┴·╜¡/╣■╢√▒⌡╩╨/└┤╫╘╣1⁄2╓┌║┼í╛┬φ┐╦╩2╛▌═°í┐_2017.docx]

各位代表：

现在，我代表市人民政府向大会报告工作，请予审议，并请市政协各位委员提出意见。

过去五年工作回顾

2012年以来的五年,是哈尔滨发展历程中不平凡的五年。在省委、省政府和市委的正确领导下，市政府紧紧依靠全市人民，认真贯彻党的十八大和十八届三中、四中、五中、六中全会精神，以习近平总书记系列重要讲话特别是对龙江两次重要讲话精神为引领，围绕“五位一体”总体布局和“四个全面”战略布局，坚持稳中求进工作总基调和新发展理念,着力稳增长、促改革、调结构、惠民生，经受住了经济下行压力带来的困难和挑战，完成了十四届人大历次会议确定的工作任务，实现了“十二五”规划圆满收官和“十三五”良好开局，开创了哈尔滨经济社会发展新局面。

——经济实现持续较快发展。经济运行保持在中高速增长合理区间，地区生产总值年均增长8%，2016年突破6000亿元大关。经济发展的质量效益稳步提升，地方公共财政预算收入同口径增加108.2亿元，年均增长7%。内需对经济增长的拉动作用显著增强，累计完成固定资产投资2.3万亿元、社会消费品零售总额1.5万亿元，分别是上个五年的2.3倍和2倍。民营经济活力加速释放，民间投资年均增长25.6%，占全市投资比重达78.1%。随着经济综合实力不断跃上新台阶，推动新一轮全面振兴的基础更加坚实，哈尔滨正朝着率先全面建成小康社会的宏伟目标加速迈进、阔步前行。

——产业转型升级取得可喜进展。五年累计开工建设2000万元以上产业项目2821个，建成投产1951个。华南城、万达文旅城等一批投资超百亿的重大项目落户我市，中船重工燃气轮机、中航科工钛产业基地、哈飞AC352直升机等众多填补国内空白的高能级产业项目相继投产见效，是哈尔滨产业项目数量最多、规模最大、质量最好的时期。食品工业产值和旅游总收入分别达到1550亿元和1039亿元，成为我市两大千亿级产业。规模以上工业增加值首次超过1000亿元，结束了副省级城市中只有我市规上工业增加值未超千亿的历史。金融、文化产业成为新的支柱产业，服务业对经济增长的贡献率由49%提高到61.3%。高新技术产业增加值由572亿元提高到1000亿元，具有核心竞争力的现代产业体系框架初步形成，为哈尔滨在新一轮产业竞争中抢占了先机、赢得了主动。

——城市功能品质大幅提升。新建改造城市道路820公里，路网体系不断完善，跨江交通更加便捷，“一江居中、两岸繁荣”发展格局基本形成。哈大、哈齐高铁正式开通，地铁1号线载客运营，3号线一期通车试运行，哈尔滨步入高铁、地铁时代。太平国际机场旅客吞吐量由2011年的784万人次提高到1627万人次，跃升东北地区机场首位。犹太老会堂、中华巴洛克等特色街区再现历史风貌，具有国际水准的哈尔滨大剧院、音乐厅、中东铁路公园成为彰显城市魅力的新地标。“哈洽会”升级为中俄博览会，哈尔滨冰雪节跃居世界四大冰雪节之首，“迷人的哈尔滨之夏”旅游文化时尚活动成为城市新的靓丽名片，成功举办国际马拉松、F1摩托艇等重大赛事，城市吸引力和影响力持续攀升，书写了哈尔滨现代化国际化城市建设新篇章。

——人民生活明显改善。城乡居民收入分别达到33190元和14438.9元，年均分别增长9.6%和12%，均高于同期GDP增速。城乡低保标准由每人每月360元和每年1350元提高到580元和3744元。企业退休人员基本养老金由人均每月1264元提高到2210元，增幅居东北地区之首。城镇累计新增就业62.8万人，年转移农村劳动力超过140万人次，被国务院评为全国创业先进城市。建成各类保障房14.36万套，改造农村泥草房、危房23万户，城乡居民住房条件明显改善。精准扶贫脱贫成效显著，139个贫困村、18.78万贫困人口实现脱贫。本届政府作出的惠民承诺全面兑现，人民群众在改革发展中有了更多获得感，城市凝聚力和向心力显著增强。

——精神文明和民主法治建设得到加强。经过三年创城攻坚，我市成功迈入全国文明城市行列。认真执行人大及其常委会的决议、决定，积极支持人民政协履行职能，自觉接受人大法律、工作监督和政协民主监督，人大代表建议、政协提案办复率始终保持100%。“六五”普法顺利完成。政府应急处置能力明显增强,成功战胜“布拉万”台风、特大洪水和暴雪等历史罕见的自然灾害，妥善处置城市供暖、道路交通和消防安全等突发事件。打造最具安全感省会城市成效显著，刑事案件发案总量连续五年保持副省级城市最低。各类安全生产事故大幅下降，被评为全国社会管理综合治理优秀城市。连续八届获评全国双拥模范城。

民族宗教、统计、审计、信访、气象、人防、地震、档案、地方志、残疾人等工作取得新进步。在经济社会深刻变革中，哈尔滨保持了政通人和、社会稳定的好局面。

各位代表，五年的发展历程让我们深切感到，办好哈尔滨的事情，必须始终坚持发展第一要务不动摇，紧紧依靠广大干部群众，以勇闯全面振兴新路子的魄力和担当，大力发扬钉钉子精神,持之以恒地抓推进、促落实。在推动改革发展的生动实践中，我们形成了一些行之有效的做法。

（一）着力抓产业上项目，持续夯实打牢全面振兴的产业根基。坚持把产业项目建设作为调结构、转动能、促发展的着力点和突破口，强化市场需求导向，推动产业建设全面提速增效。一是突出规划引领。立足资源禀赋和产业基础，科学编制全市“4+6+1”产业发展规划，进一步明晰各区县（市）主导产业和园区定位，形成了错位发展、优势互补的产业建设新格局。二是强化创新驱动。采取政校企共同参股和股权激励模式，组建省工研院、市食研院等新型研发机构，携手哈工大等大学大所创建6大科技成果转化平台,累计转化成果2324个。深入实施科技创业三年行动计划，全市新增科技型企业2075户、国家级科技企业孵化器22家，安天科技、哈工大机器人等高技术企业成为行业龙头。我市入选全国首批小微企业创业创新基地示范城市。三是加快园区建设。累计投入园区发展专项资金30亿元，提升园区产业承载和集聚功能。经开区、高新区、利民和宾西开发区已初步形成高端装备制造、绿色食品、生物医药、现代包装、大数据等产业集群。巴彦等4个县域工业园区晋升为省级开发区。四是扩大招商引资。开展全域产业大招商，构建了以各区县（市）、各开发区为主体，市直部门齐参与的招商新格局。累计引进开工产业项目2036个，实际使用内资7525亿元、外资131亿美元，分别是上个五年的3.6倍和4.2倍。五是保障要素供给。支持企业通过资本市场直接融资，在境内外新上市、挂牌企业62家，是2011年以前上市、挂牌企业总和的2.5倍。累计发行企业债券72.9亿元，争取国家专项建设基金139.8亿元。市企业信用融资担保服务中心担保额达616亿元，政府创投引导资金撬动社会出资38亿元，哈尔滨成为全国创业融资排行榜东北地区唯一上榜城市。加强人才和用地保障，累计引进各类产业发展急需人才8000余人，新增用地规模111平方公里，更好满足了产业建设需求。六是创优发展环境。出台稳增长促发展、推进供给侧结构性改革、促进民间投资等一系列产业扶持政策，实施产业项目全周期管理和全过程服务，累计帮助企业解决难题670件，为企业减税降费超过70亿元。南岗、道里连续三年入选全国投资潜力百强区。

五年的实践证明，产业项目建设牵动全局、引领发展，必须以功成不必在我的胸怀，一届接着一届抓，一任连着一任干，大力推动产业不断迈向中高端水平，为打赢新一轮老工业基地振兴攻坚战提供强大的动力引擎。

（二）着力统筹城乡一体化发展，持续释放县域经济活力。积极抢抓两大平原现代农业综合配套改革机遇，全面提高农业综合生产能力和比较效益，市财政累计投入57.7亿元，推动县域经济步入科学发展轨道。一是加快促进农业生产方式转变。不断加强农业机械化、水利化、科技化、生态化建设，综合机械化率由90%提高到95%，农田有效灌溉面积由477万亩扩大到1110万亩，现代农业示范园区（带）核心区面积发展到1556万亩，以“五常大米”为代表的绿色、有机食品认证面积增加到1282万亩。年均粮食产量达339亿斤，连续四年成为全国第一产粮大市。国家级标准化畜禽养殖场达34家，肉蛋奶总产量保持在300万吨以上，稳居全国副省级城市首位。二是深入实施产业强县工程。坚持以“粮头食尾”、“农头工尾”为抓手促进县域工业化。市级以上重点龙头企业发展到195家，龙良油脂三期、海通食品电商平台等580余个项目陆续建成投产，巴木通方依产业一体化初具规模。完成县域规上工业总产值超过6900亿元，是上个五年的3.8倍。三是不断深化农村产权制度改革。率先在全省开展农村土地确权登记颁证工作，提前三年完成国家确定的改革任务。率先在全省启动集体资产股份权能改革试点。积极推进土地规模流转，新型农业经营主体发展到1.5万余个，土地规模经营面积扩大到1790万亩，是确权前的3.7倍。四是扎实推进新型城镇化建设。累计撤并乡镇17个、村屯700个，新建新型农村社区100个、美丽乡村200个，20余万农民实现了向城镇和新型农村社区有序转移。新建农村饮水安全工程2691处、农村公路4236公里。木兰松花江公路大桥建成通车，结束了两县之间靠摆渡过江的历史。

五年的实践证明，统筹城乡发展是新常态下扩内需稳增长的客观要求，只要我们坚定不移地走农业产业化、县域工业化、区域一体化的发展之路，就一定能够不断开创城乡和谐发展、共同繁荣的新局面。

（三）着力加强城市规划建设管理，持续提升城市承载力和吸引力。深入贯彻中央城市工作会议精神，创新城市建设理念和管理模式，加快现代化国际化城市建设步伐。一是科学规划引领城市发展。开展城市总体规划修编，完成新型城镇化、哈尔滨新区、海绵城市等规划200余项，以“十大支撑性规划”为框架的规划体系基本形成,为进一步优化城市空间布局，塑造哈尔滨大气洋气神气的城市风格奠定了坚实基础。二是统筹建设提升城市功能。累计投入2170多亿元，新建改造道路468条、桥涵120座。沿江景观大道、三环路江南江北实现贯通，进乡街高架桥、保健路地道桥、和谐大道跨线桥建成通车，城市现代化交通体系逐步完善。新建改造源网厂站67座，改造各类管网3100公里，地下综合管廊建设进度居全国首批10个试点城市之首，“智慧管网”地理信息系统投入使用，城市运行保障能力显著提升。新改扩建停车场92处、公交场站94座，新增大容量环保公交车3500台，“公交都市”创建工作始终走在全国前列。三是精细化管理规范城市运营。推动管理重心下移，强化区街主体责任，主干街路环卫机械化作业实现全覆盖，主城区垃圾密闭运输率达100%。持续加大环境综合整治力度，创建环境卫生达标街路280条，改造老旧居民庭院1454个，清理违规牌匾广告86万余处，违法小广告、沿江岸线船舶乱停乱靠等城市顽疾得到有效治理。重拳打击违建蔓延歪风，拆除各类违法建筑2.4万处、950.8万平方米，有力地维护了社会公平正义。坚定实施绿色清冰雪，确保了冬季城市运行通畅和环境整洁，彰显了冰雪城市的特色魅力。四是从严管控改善城市生态环境。编制环境总体规划，初步划定生态保护红线区2.3万平方公里。深入落实国家大气、水污染防治行动计划，拆并10吨及以下燃煤锅炉2740台，淘汰黄标车及老旧车10.2万辆，秸秆综合利用率提高到63%。2016年空气质量优良天数达279天，比上年增加52天。新增城镇污水处理厂11座，城市污水集中处理率提高到91%。城市内河治理取得重大突破，马家沟实现清水入河，何家沟治理工程荣获中国人居环境范例奖。人工造林53.7万亩，城区新植树木255万株，新增绿地688公顷，人居环境得到明显改善。

五年的实践证明，持续提升城市承载功能和形象品质是顺应人民群众期待、增强城市竞争力的现实选择，只要我们始终坚持以人为本，推动城市规划建设管理全方位升级，哈尔滨就一定会更加和谐宜居、更富发展活力。

（四）着力深化重点领域改革，持续增强经济发展的内生动力。突出问题导向，打破利益固化藩篱，下决心破除制约发展的体制机制弊端。一是大力推进简政放权。率先在全国有立法权的城市中取消所有市级自设的行政审批事项，建设项目审批时限平均压缩50%以上。首批入驻市民大厦的510项涉企行政审批事项实现一条龙服务、一站式办结。率先在全省推行政府部门权责清单和审批中介服务清单制度，政府事权精简52.5%，相关中介服务事项减少35%。率先在全省完成市场监管等“大部门制”改革，实施以整合优化部门内设机构为重点的“大处室制”改革，有效解决多头管理、推诿扯皮等问题，得到了中央深改办的充分肯定。率先在全省实现企业登记“五证合一、一照一码”，有力地激发了“双创”活力，全市各类市场主体达61万户，比改革前增长85%。二是稳步推进国资国企改革。推动市属国有企业与行业管理部门脱钩，整合组建交通、建投等9大集团，带动市属企业国有权益总额、收益额持续提升，分别是2011年的1.5倍和3倍。通联客车完成混合所有制改革并运营投产，哈药集团完成人民同泰和三精制药资产整合，哈投股份、江海证券通过重组实现股本规模和券商评级“双提升”，物业供热集团在我市首单获批发行国有企业资产证券。在哈中直省属企业“三供一业”分离移交工作基本完成。三是积极推进公共资源市场化配置。开展政府与社会资本合作，轨道交通、地下综合管廊等9个项目累计引入社会资本483亿元。建立国有资产进场转让租赁机制，先后拍卖地铁灯箱广告等国有资产经营权，累计成交额达18.7亿元。四是加快推进财税管理机制创新。进一步完善市对区县（市）财政管理体制，深化预算公开、第三方绩效评价等改革。公共财政累计支出3795.2亿元，年均增长9.5%，财政支撑能力显著增强。通过争取置换债券资金、建立政府债务风险预警机制等措施，有效防控政府债务风险、降低财务成本。落实营改增政策，创新税收管理办法，税收占财政收入比重保持在80%左右。

五年的实践证明，面对经济发展新常态，我们唯有全面深化改革，正确处理好政府与企业和市场的关系，才能从根本上解决多年积累的深层次矛盾，让哈尔滨在新一轮老工业基地振兴中不断焕发出蓬勃生机。

（五）着力推进对外开放合作升级，持续扩大经济发展的外向度。抢抓“一带一路”战略“龙江丝路带”建设的历史机遇，扎实推进对俄合作中心城市建设。一是建设互联互通大通道。先后开通11条对俄客运和货运包机航线，获批运行东北首条第五航权航线，龙运哈俄公路通道正式开通，哈欧班列保持每周双向对开，哈俄班列、“哈绥符釜”陆海联运实现常态化运营，哈尔滨国际集装箱中心站和北美进口快件监管中心投入使用，对俄邮政小包裹发运量稳居全国首位，构筑起了西接欧洲、东接东北亚乃至北美的便捷货运通道。二是打造对外开放大平台。综合保税区一期顺利建成，上海东浩兰生保税物流、德国豪狮农机装备制造等24个项目签约入驻。临空经济区的大飞机拆解项目已完成主体工程，跨境电商物流基地等项目进展顺利。哈尔滨新区涉及的三个行政区已先行完成与相应开发区的统一管理改革，按照负面清单管理模式建立的市场准入审批系统上线运行。哈以合作上升为国家战略，中以国际航空产业园、现代农业产业园进入实质性推动阶段。中白产业园一期核心区基础设施全面建成，莫斯科俄速通海外仓投入运营，中俄农业产业示范园启动建设。三是拓展对外合作新领域。以对俄合作为重点，不断扩大对欧美俄日韩等地区贸易往来，累计完成进出口总值236.4亿美元，是上个五年的1.25倍。成功举办对俄音乐互访演出、艺术展示、科技交流等活动近百场，成立中俄跨境金融合作联盟。太平国际机场实现72小时过境免签，获批全省首批离境退税试点城市，建立叶卡捷琳堡境外旅游营销中心，对外开放的广度和深度不断拓展。

五年的实践证明，扩大对外开放是推动我市全面振兴发展的重要途径，只要我们立足现有优势，加快打造跨境产业链和产业集聚带，积极推进全方位对外合作升级，就一定会在国家和省对外开放战略实施中把握机遇、乘势而上、大有作为。

（六）着力保障和改善民生，持续增进人民群众福祉。全市财政用于民生支出占总支出的70%以上，带动惠民行动项目投资超过1320亿元。一是大幅提高社会保障能力。稳步推进机关事业单位养老保险制度改革，基本养老保险实现政策全覆盖。在全省率先实施城乡居民大病保险制度，启动城镇基本医疗保险市级统筹，新农合统筹区域内政策性报销比例提高到78.4%,异地就医即时结算业务走在全国前列。发放各类社会救助金69亿元，惠及城乡困难群众达274.7万人次。全市各类养老机构床位由2011年的3.2万张增加到4.8万张，城市社区日间照料中心和居家养老服务站实现全覆盖。二是妥善解决历史遗留问题。物业管理、冬季供暖等群众反映强烈的问题得到有效解决。创建市级以上物业管理示范小区354个，维修老旧住宅2284万平方米。新增集中供热能力8192万平方米，居民供热投诉量下降50%。道外桥头屯和新一地区1.5万户内涝居民全部得到避险搬迁安置。三是不断提升基本公共服务水平。新改扩建公办幼儿园268所、中小学校65所，创建素质教育特色学校91所、职业教育实训基地47个，组建义务教育集团化联盟70个，外来务工人员随迁子女全部就读公办学校。基本公共卫生人均补助经费由25元提高到45元。道里、方正“云医疗”信息化平台建成使用。市第一医院改革成效明显，县级公立医院及所有基层医疗机构全部取消药物加成。哈夏音乐会等群众性文化活动影响力不断扩大，国际油画展示交易中心、“731”遗址新馆、市规划展览馆等一批文化馆所建成开放，城乡公共文化服务网络和城区健身设施实现全覆盖。

五年的实践证明，抓民生也是抓发展，保障和改善民生没有终点站，只有连续不断的新起点，必须持之以恒、常抓不懈，努力走出一条基本民生得到改善、百姓福祉不断提高、小康社会全面实现的新路子。

（七）着力加强政风建设，持续提高政府办事效率和服务水平。认真开展党的群众路线、“三严三实”、“两学一做”等专题学习教育，推进依法行政、从严治政。一是强化行政问责。先后制定出台行政问责、规范涉企执法行为、行政执法人员“六项禁令”、执法检查“双随机、一公开”等制度规定，累计查处吃拿卡要、失职渎职等破坏经济发展环境案件1083起，问责2047人，有效地教育和警示了广大公职人员。二是强化社会监督。设立企业投诉受理中心，建立企业评议、执法监督等工作机制，累计召开7次政风建设大会、5次现场评议活动，企业对行政审批和执法的满意率由2012年的76%提高到82%。三是强化督办落实。坚持言必信、行必果，严格执行绩效评估和督办检查工作制度，市政府跟踪督办事项达6000余件，推动重大决策事项和重点工作任务一抓到底、落实到位。

五年的实践证明，实现全面振兴，队伍建设是关键，必须坚持不懈地改进政风，努力增强广大公务人员的责任意识和服务意识，切实提高服务经济社会发展的能力和本领，才能汇聚起谋发展促振兴的强大合力。

各位代表，过去五年的成绩来之不易，这是省委、省政府和市委正确领导、科学决策的结果，是全市广大干部群众团结一心、拼搏奋进的结果，是人大代表、政协委员和社会各界携手努力、鼎力支持的结果。在此，我代表市人民政府，向所有关心支持哈尔滨事业发展的同志们、朋友们，向广大驻哈解放军指战员、武警官兵，致以崇高的敬意和衷心的感谢！

在充分肯定成绩的同时，我们也清醒地认识到，发展中还存在不少困难和问题：经济总量规模小、财政收支压力大，经济综合实力亟待增强；产业转型升级步伐还不够快，工业比重偏低、战略性新兴产业发育不足仍是制约我市振兴发展的最大短板；支持创新创业的资金、人才和制度等要素保障体系还需完善，内生动力和活力有待进一步释放；市场化进程相对迟缓，公共资源和产品的市场竞争不够充分，民营经济发展水平还需提高；保障民生和社会治理能力不足，公共服务、社会事业发展和环境污染治理等方面群众不满意的问题仍然存在；政府少数工作人员法治意识、规则意识、创新意识、服务意识不强，一些领域的不正之风和腐败问题还时有发生。我们要下决心解决这些问题，全力做好政府各项工作，不负人民重托。

今后五年及2017年工作

今后五年是全面建成小康社会的决胜期，也是哈尔滨全面振兴发展爬坡过坎的攻坚期。刚刚胜利闭幕的市第十四次党代会，客观分析了今后五年我市面临的发展形势，科学规划了全面建成小康社会的宏伟蓝图，明确提出了推动全面振兴发展的战略任务。今后五年政府工作的指导思想是：全面贯彻党的十八大、十八届三中、四中、五中、六中全会和即将召开的党的十九大精神，深入贯彻习近平总书记系列重要讲话特别是对我省两次重要讲话精神，统筹推进“五位一体”总体布局和协调推进“四个全面”战略布局，全面贯彻落实新发展理念，认真落实省委省政府“五大规划”、“十大重点产业”、“龙江丝路带”等战略部署，坚持稳中求进工作总基调，以提高发展质量和效益为中心，全面做好稳增长、促改革、调结构、惠民生、防风险各项工作，奋力走出一条质量更高、效益更好、结构更优、优势潜能得以充分释放的全面振兴发展新路子。

今后五年的奋斗目标是：到2020年，地区生产总值和城乡居民收入比2010年翻一番，如期全面建成小康社会。力争今后五年，地区生产总值年均增长6.5%左右，公共财政收入年均增长4.5%，经济综合实力和民主法治、文化发展、民生福祉、绿色发展水平显著提高。为实现上述目标，工作中我们要把握好以下五个方面：

——坚持创新发展理念，切实增强全面振兴发展新动能。把振兴发展的基点放在创新上，主动顺应速度变化、结构优化、动能转换新趋势，突出供给侧结构性改革主线，扎实做好改造升级“老字号”、深度开发“原字号”、培育壮大“新字号”三篇大文章，着重在工业扩量升级、服务业提质增效、大众创业万众创新等重点领域，实施一批牵动性产业项目和关键性改革措施，打造经济增长新引擎。力争用五年时间，使科技进步对经济增长贡献率提高到65%以上，战略性新兴产业和现代服务业比重大幅提升，高端装备制造、新一代信息技术跨入千亿级产业行列，汽车、民用航空、燃气轮机、智能装备及机器人、新材料及3D打印等具有较强竞争力的产业集群基本形成，经济社会发展的质量和效益明显提高。

——坚持协调发展理念，不断提升区域整体发展水平。积极抢抓哈长城市群发展机遇，适应城市化进程中出现的逆城市化新特征，统筹城乡规划建设和产业布局，有序推进新型城镇化建设，着重在完善城镇综合承载功能、推动农村转移人口市民化、促进区域经济协调发展等方面实现新突破。力争用五年时间，户籍人口城镇化率达到55%。机场扩建、哈站改造、阳明滩北部联络线等重大区域联通工程竣工投用，主城区与新城区及周边卫星城交通网络更加完善，巴木通方依区域一体化发展更趋紧密。哈长城市群和哈尔滨都市圈实现产业优势互补、经济互动共赢，区县协同、产城融合、城乡一体、区域联动发展的新格局基本形成。

——坚持绿色发展理念，加快建设美丽生态宜居哈尔滨。深入实施大气、水、土壤污染防治行动计划，重点在城乡垃圾污水处理和环境综合治理、冬季清洁取暖、绿色建筑推广、沿江景观带保护开发、发展循环经济、企业达标排放等方面实施一批基础性工程，强化生态损害责任追究，确保生态环境质量总体改善。力争用五年时间，主城区基本淘汰分散燃煤小锅炉，秸秆综合利用率超过85%，城市环境空气质量达标率达到80%以上，市区建成区基本消除黑臭水体，阿什河水质达到或优于五类标准，森林覆盖率稳定在46%以上，单位生产总值用水量、能耗、二氧化碳排放量大幅下降，让哈尔滨的天更蓝、地更绿、水更清、空气更清新。

——坚持开放发展理念，着力构建开放型经济新体制新格局。深度融入国家“一带一路”战略，积极落实省“龙江丝路带”建设部署，重点畅通航空、铁路、公路等国际物流大通道，巩固提升哈尔滨新区、综合保税区、临空经济区、中以和中白产业园等平台的产业集聚功能，引进建设一批支撑性强、外向度高的产业大项目，积极申建哈尔滨自贸区，全面推行准入前国民待遇加负面清单管理制度，加快形成法治化国际化营商环境。力争用五年时间，构建起面向俄罗斯和东北亚、承接欧美地区更加便捷的跨境运输通道体系，加工贸易、服务贸易和跨境电子商务比重显著提升。将哈尔滨新区建设成为中俄全面合作重要承载区和东北地区体制最新、效率最高、功能最优的新增长极，使哈尔滨对俄合作中心城市地位更加巩固、作用更加凸显。

——坚持共享发展理念，持续提升群众获得感和幸福感。始终秉持以人民为中心的发展思想，着力解决好人民群众普遍关心的突出问题，进一步巩固提升文明城市创建成果，促进创业带动就业，稳步提高社会保障和公共服务水平，坚决打赢精准脱贫攻坚战。力争用五年时间，实现城镇新增就业51.5万人以上，城乡低保、最低工资、养老保险待遇等标准明显提高，全面建立城乡统一的居民基本医疗保险制度。市图书馆、群众艺术馆和奥体中心等重大文体设施建成投用。二环以内及三环、四环周边棚户区改造基本完成。延寿、木兰、巴彦3个贫困县全部摘帽，现行标准下的贫困人口全部脱贫，努力让改革发展成果更多更公平地惠及全体人民。

各位代表，今年是新一届政府的开局之年，是实施“十三五”规划的重要一年，也是供给侧结构性改革的深化之年，做好今年工作意义重大。我们要牢记习近平总书记的重要嘱托，坚持扬长避短、扬长克短、扬长补短，全面落实“四个着力”、“三篇大文章”、“五头五尾”、“五个要发展”等重要工作部署，更加注重产业转型升级和振兴实体经济，深入落实“三去一降一补”任务，持续推动经济平稳健康发展和社会和谐稳定，更好发挥省会城市龙头带动作用。综合考虑各方面因素，今年经济社会发展的主要预期目标是：地区生产总值增长7%左右；固定资产投资增长7%以上；规模以上工业增加值增长5%左右；社会消费品零售总额增长10%左右；地方公共财政预算收入增长3.5%以上；城镇和农村居民人均可支配收入增长7%左右；城镇登记失业率控制在4.3%以内。

为实现上述目标，建议做好以下八项工作：

（一）坚持把供给侧结构性改革的着力点放在振兴实体产业上，进一步提高经济发展质量和支撑能力。着重在既有市场需求又有供给优势的产业领域精准发力、科学施策，加快形成消费与供给良性互动、需求与产业协同升级的发展格局。一是做大做强工业经济。牢牢把握新型工业化发展方向，积极争创国家“中国制造2025”示范城市，深化制造业和互联网融合发展，加快推进东安汽车D系列发动机、哈电集团核主泵机组等80个亿元以上项目建设，推动长安福特汽车、哈锅生产基地等项目尽快投产见效。主动适应食品消费升级新趋势，重点抓好哈工大中奥花青素、百威英博啤酒二期等项目建设，推动食品工业加速向2000亿元产业迈进。围绕抢占新产业和新业态竞争高地，积极推进中国移动数据中心、圣泰生物制药、中博新能源、中大路桥装配式建筑等重点项目建设提速。主动承接央企技术外溢，合作盘活闲置资源，突出抓好东轻铝镁合金产业园、哈飞直升机等37个与央企合作项目建设。二是促进旅游业创新升级。积极推进旅游与文化时尚、体育健康、养生养老等产业融合发展，把冰雪、湿地、森林、音乐、绿色食品等方面优势资源变成有竞争力的产品和服务。重点建设运行好万达文旅城、波塞冬海洋王国、呼兰河口湿地、西城红场、新海都国际温泉旅游城、奥体中心一期等重大项目，切实提高哈尔滨大剧院、音乐厅等场馆市场化运营水平，在重点旅游景区开展特色鲜明的驻场演出，在观赏游玩、文化创意、养生健身、休闲度假中创新服务供给、拉长消费链条。进一步扩大“冰雪节”、“迷人的哈尔滨之夏”等节庆活动和城市马拉松、帆船等重大赛事的国际影响力，带动产业集群发展。更加注重市场营销和产品设计，持续叫响旅游文化时尚名城品牌。三是加快发展生产性服务业。充分发挥道里、南岗、松北、哈南等金融创新区服务实体经济作用，支持三联药业、森鹰窗业等多家企业上市融资，哈尔滨股权交易中心新增挂牌企业300家。大力发展现代物流、电子商务、商贸会展、科技服务等生产性服务业，积极推进国家现代物流创新发展试点城市建设，鼓励传统商业拓展线上线下销售，加快建设传化智能公路港、人和集团地利农产品交易中心、阿里巴巴哈尔滨产业带等重大项目，推动生产性服务业向专业化和价值链高端延伸。四是加速园区产业集聚。经开区重点打造航空汽车、大数据等功能区，确保中国电信数据中心等30个产业项目按期开复工。高新区重点抓好航天产业园、碧海产业园、电子信息产业园和科技孵化园建设，新增科技类企业48家。利民开发区突出抓好恒泰云商产业城等产业项目，确保圣泰小牛血等项目建成投产。宾西开发区重点推进烟叶复烤等项目建设，力争建成投产5个以上。五是提升招商引资效益。探索建立政府购买服务和多元化招商鼓励制度，增加对重点产业、新兴产业、服务贸易的考核权重，注重引资引技引智并举，更多引进世界500强、行业领军企业和带动作用突出的大集团、大项目，为产业转型升级不断注入新的强大动力。

（二）突出创新驱动和质量引领，进一步提升实体经济的核心竞争力。坚持把创新作为实体产业发展的制胜法宝，加快建设质量强市，着力扩大高质量产品和服务供给。一是强化应用技术研发。投入应用技术研究与开发资金2.65亿元，支持在高端装备、智能机械、增材制造等领域推进一批重大科技成果转化项目。进一步落实高新技术企业认定资助、研发平台建设支持等政策，促进企业更多研发新产品、形成新产业。二是打通科技成果转化通道。以创新园、创业园为载体，进一步加强与在哈高校、科研院所合作，加快推进职务科技成果转化处置权、使用权和收益分配权改革，对校所科技人员科技创业项目给予政策性股权投资支持，打开科研人员在事业单位和企业间流动的“旋转门”，激励更多科技人才创新创业，落地转化科技成果300项以上。三是推动大众创业万众创新。加快推进哈尔滨新区争创国家自主创新示范区和双创示范基地，积极引进京东云创空间、阿里云创业大学等创业机构，为科技人员和大学生创新创业提供优质服务。上下联动打造大直街校所经济带，支持龙门科技、创投孵化器集团做大做强，市级以上科技企业孵化器突破70家，新增在孵企业350家。启动实施哈尔滨英才集聚计划，新建市级领军人才梯队10个、博士后双创实践基地15个。推进科技创新人才专项资金项目和创业创新券项目400个，进一步扩大科技风险投资基金规模，不断放大科技创新与双创协同发力的乘数效应。四是深入开展质量提升行动。加强全面质量管理，引导企业牢固树立质量第一意识，发扬工匠精神，重点在装备制造、绿色食品、新型建材和云计算大数据等领域，着力增品种、提品质、创品牌，不断提高本地企业产品市场竞争力和占有率。

（三）毫不放松抓好“三农”工作，进一步壮大县域经济整体实力。深入推进农业供给侧结构性改革，促进农业由主要满足量的需求向更加注重满足质的需求转变，加快释放县域经济发展潜能。一是推动农业生产调结构增效益。把增加绿色优质农产品供给放在突出位置，坚持“稳粮、优经、扩饲”，依托五常、双城打造国家级水田、旱田现代农业示范园区，建设呼兰、阿城、宾县、双城、五常等5个“北菜南运”示范区县，“互联网+农业”高标准农田示范基地发展到80个。积极推进松花江沿岸灌区“旱改水”和通河二甲沟、依兰丹青等重大水利工程建设。实施农业“三减”面积300万亩、黑土地保护项目20万亩。深入推进“两牛一猪一禽”规模化养殖，肉蛋奶产量保持在300万吨以上。健全质量安全监管追溯体系，探索推广农产品点对点、特色定制、网上专卖等营销新模式，继续扩大五常大米、巴彦生猪、尚志黑木耳等知名品牌影响力，推动“种得好”向“卖得好”转变，带动倒逼“种得更好”。二是深入实施产业强县工程。坚持市场导向，依托资源优势，以精深加工为重点发展县域主导产业，促进“原字号”多次加工、梯次增值。加快推进万润百万头生猪屠宰加工、伟业国际木材产业园、奥瑞德纳米陶瓷部件等项目建设，重点培育五常金福泰、木兰昊伟等50家市级龙头企业，着力打造巴木通方依一体化特色产业带，构建各具特色的县域经济发展新格局。三是深化农村综合改革。积极推进三权分置改革，深入开展方正土地承包经营权和宅基地使用权抵押贷款试点，启动100个村集体经济组织股份权能改革，赋予农民更加充分的财产权利。推动新型农业经营主体向一二三产融合方向发展，新增市级以上农民专业合作社规范社120个。创建哈尔滨粮食交易所，深化供销社综合改革，逐步构建起以企业和经营性组织为核心的服务“三农”支撑体系。四是扎实推进新型城镇化和美丽乡村建设。继续做好10个国家和省重点镇综合改造。依托综合保税区、临空经济区等重点产业集聚区，谋划启动一批宜居宜业、产城融合的特色小镇。大力整治农村人居环境，推广方正县村镇垃圾处理经验，创建美丽乡村100个，集中整治自然屯1000个，解决51个贫困屯2.25万人饮水安全问题，开工建设农村公路300公里，为促进农业增效、农民增收和农村繁荣创造更好条件。

（四）继续抓好城市规划建设管理，进一步提升城市功能品质。突出城市修补和生态修复，努力让群众生活得更安心、更省心、更舒心。一是加强规划指导。落实和评估2020年城市总体规划，推广“多规合一”模式，科学编制哈尔滨新区、综合保税区等控详规划，切实提高规划编制的科学性、操作性和规划执行的严肃性。二是完善城市功能。围绕满足重点产业项目、园区发展需求和打通交通堵点，开工建设路桥工程30项，实施地下综合管廊二期工程，统筹推进地铁1号线三期、2号线一期、3号线二期建设，启动4号线、5号线，积极配合哈站改造、哈佳快速、哈牡客专和哈尔滨机场扩建工程加快建设，确保滨北铁路桥建成通车。三是强化城市管理。发挥“大城管”机制作用，推动市、区、街一体化管理，城区道路机械化清扫、水冲洗作业率和机械化清冰雪率均提高到80%以上。突出抓好城乡结合部、市场摊区等环境卫生整治工作，继续保持打击违建高压态势。加快推进松北呼兰、阿城生活垃圾焚烧项目以及市危险废物综合利用处置项目建设，确保双城垃圾处理场投入使用、餐厨废弃物处理厂试收运，城区生活垃圾无害化处理率达到91.6%。推进智慧城管云数据中心及配套平台建设，为打造智慧城市创造条件。四是加强环境治理和生态保护。全面完成生态保护红线划定，深入落实大气、水污染防治行动计划，着力解决中央环保督察组反馈的突出问题。进一步加大10吨及以下燃煤锅炉和黄标车淘汰力度，秸秆综合利用率提高15个百分点以上。大力推进建成区黑臭水体整治，确保松花江干流哈尔滨段水环境质量达到国家考核标准。全年人工造林3.8万亩，城区新植树木10万株，新增绿地11.7公顷，让广大市民的生活环境更加生态宜居。

（五）深入推进重要领域和关键性改革，进一步激发市场活力和社会创造力。围绕供给侧结构性改革要求，以经济体制改革为重点，按照“四个有利于”标准，着力破除体制机制障碍，释放更多改革红利。一是深化行政体制改革。对重点工业及仓储类项目、基础设施及公益类投资项目，实行分阶段并联审批和集中联合会审，大力推行多评合一、多审合一、多图联审、联合验收等新模式，进一步降低制度性交易成本。动态调整权责清单，推行公共服务事项清单制度。开展“减证便民”专项行动，对适合网上办理的事项一律开展线上服务，真正做到让信息多跑路、群众少跑腿。有序推进事业单位分类改革，推动行业协会商会与行政机关全面脱钩，促进人力资源、社会资源向创造财富的生产部门配置。二是大力推进国资国企改革。推动水投集团等平台类公司加快向商业类转型，充分参与市场竞争。组建商贸物流集团、人防集团，进一步提高国有资产配置效益。加大混合所有制改革力度，积极推动电碳厂与法国梅尔森、松江电机与广联航空改制重组，力争市属国有集团二级企业混改取得突破性进展。大力推动市场化选聘职业经理人，国有出资企业外部董事实现全覆盖。更多运用市场化、法治化办法，加速清理空壳和僵尸企业。全面完成厂办大集体改革补漏工作。三是创新财税管理体制。实施跨年度预算平衡机制，推进政府资产、公共资源市场化运营，大力推广运用PPP、政府购买服务、股权投资等财政资金投入方式，进一步健全政府债务风险防控体系，确保财政资金安全规范高效使用。充分发挥税收职能作用，不断提高税收征管和服务水平。四是加快发展非公经济。进一步放宽交通、市政公用、社会服务等领域市场准入，在财税优惠、土地供应等方面一视同仁，鼓励民营企业扩大投资、参与国企改制重组。加快设立中小企业发展基金，支持企业通过应收账款、知识产权质押等方式拓宽融资渠道。全年非公经济增加值增长8%左右。

（六）积极扩大对外开放,进一步在开放中增创竞争新优势。扎实推进对俄合作中心城市建设,拓展欧美、日韩及拉美市场，推动“南联北开”取得更大成效。一是立足区位优势拓通道。充分发挥太平机场国际航空枢纽功能，引入川航、深航开辟北美航空货运通道，扩大哈欧、哈俄班列常态化运营规模。重新启用哈尔滨水运口岸。积极申建中俄跨境电商综合试验区，做大对俄电商小包规模，加快形成面向俄罗斯、辐射东北亚、联通北美的国际物流大通道。二是立足开放平台引项目。综合保税区突出抓好“港区一体化”及海关多式联运监管中心建设，积极引进优传保税仓储展示、坤德物流等企业进驻。临空经济区重点推进跨境电商物流基地、哈尔滨航空邮件处理中心等项目建设，确保大飞机拆解项目投产运营。

中以产业园采用“一区多园”模式，加快推进国际航空产业园、现代农业产业园等项目落地建设。三是立足密切交往促合作。进一步扩大与美俄日韩及大洋洲的经贸往来，切实发挥“中俄博览会”、“寒博会”等国际化品牌展会作用，深化与俄罗斯、白俄罗斯等国家的科技交流合作，设计开辟哈尔滨至莫斯科、圣彼得堡、叶卡捷琳堡等精品旅游线路，打造中俄跨境金融中心，开展油画、动漫等艺术展演展示活动，办好第二届“中俄文化艺术交流周”。积极推动与深圳对口合作取得实质性进展。

（七）全面推进哈尔滨新区建设，进一步打造全省改革开放创新发展样板区。充分发挥国家级新区先行先试优势，坚持治理结构扁平化、行政审批清单化、要素配置市场化、产业项目高端化，推动哈尔滨新区提速建设。一是推动体制机制创新。全面实行大部门制和扁平化管理体制,推进街道管理体制改革，推动管理层级压缩、重心下沉。率先在条件成熟的功能区开展企业化管理试点，在公共资产运营、人才引进选用、投融资等领域实行市场化、社会化运作，不断激发新区发展活力。二是打造与国际接轨的一流营商环境。全面推行“非禁即入”的负面清单管理模式，建立“统一规范、并联运行、容缺受理、信息共享、阳光操作、全程监督”的审批监管体系，切实做到企业投资办事“审一次”、“不出新区”。不断完善综合执法、相对集中行政处罚等监管机制，营造公平、透明、可预期的投资环境。三是启动建设一批先导性支撑性项目。按照“多规合一”新区规划体系，发挥国家服务贸易创新发展试点等政策集成优势，围绕新技术、新产业、新业态、新模式招大引强上项目。进一步强化产业项目全过程服务，突出抓好林达外语城、空间环境地面模拟装置等项目建设，加快建设万鑫石墨谷、星忆新型存储芯片、博睿创富航空航天高纯金属材料、中丹智能供热装备制造等高技术产业项目，确保哈工大卫星激光通讯、航天海鹰3D打印钛合金制粉等项目建成投产。坚持向上争取、市场融资多措并举，加快建设浦源路、中心南北大道等交通干道，以及城区松花江水源供水工程、呼兰老城区污水处理二厂等公用设施，启动实施科技创新城二期工程建设，不断提升新区基础设施的综合承载力。

（八）不断加大保障和改善民生工作力度，进一步让群众共享改革发展成果。重点实施70个惠民行动项目，完成投资185亿元，着力织密筑牢民生保障安全网。一是强化精准扶贫。进一步夯实延寿国家级贫困县脱贫基础，实现巴彦、木兰两个省级贫困县摘帽，46个贫困村、41276名农村贫困人口脱贫。二是坚持创业带动就业。努力为科技人员、大学生、农民、城镇转移就业职工提供更多的创业机会，促进更多人力资源进入新领域、新业态，特别是以“互联网+”为基础的电商、微商、众筹等新商业模式，实现自主创业。全年城镇新增就业10.3万人，推动农民创业17万人。三是改善城乡居民住房条件。综合运用合理调控土地供给、扩大棚改货币化安置、发展住房租赁市场和跨界地产等措施，扎实推进房地产去库存。开工建设棚改住房7000套，为1.7万户困难家庭提供住房保障。改造农村危房30万平方米。维修老旧住宅70万平方米，创建物业管理示范项目30个，不断提升物业管理服务水平。四是优先发展公共交通。积极发展定制公交、社区公交、智能公交，开辟调整延伸公交线路12条，更新公交车辆600台，启动102国道哈尔滨至双城段拓宽改造工程，努力让百姓出行更便捷。五是完善社会保障体系。扎实做好机关事业单位养老保险制度改革工作，继续实施助保贷款政策。稳步推进城镇居民医保与新农合制度整合，持续推进基本医疗保险市级统筹，新农合人均筹资标准提高到630元。进一步增加医保异地结算机构数量，实现异地就医即时结报。适时提高城乡低保标准，切实保障困难群众基本生活。深化国家养老服务业综合改革试点，积极培育规模化、连锁式养老机构，全年新增养老床位3000张。六是努力办好人民满意教育。加固维修学校20所，新建中小学校8所，创建义务教育特色学校15所，义务教育学校基本达到标准化。深入实施集团化联盟办学，鼓励发展民办教育，持续扩大优质教育资源覆盖面。加快启动市医药、食品两个职教集团组建工作，探索建立校企合作、工学结合、产教融合的现代职业教育新模式。七是加快打造健康哈尔滨。统筹推进城市公立医院综合改革，大力发展“云医疗”和家庭医生签约服务模式，加快建立分级诊疗制度，组建医疗联合体50个。提高突发公共卫生事件应急能力和妇幼健康服务能力，免费为城乡居民提供13类46项基本公共卫生服务。八是繁荣发展文化体育事业。积极开展星级文明户、文明村、文明乡（镇）等创建活动，不断提高市民文明素质。加快推进国家文化消费试点城市建设，支持道里争创第三批国家公共文化服务体系示范区。继续推进“731”、金上京申遗工作，广泛开展农民艺术节、社区文化节、“全民上冰雪”等群众性活动，打造社区百姓健身屋80个以上，为丰富群众文体生活创造条件。九是加强社会治理。深入实施“强基工程”，新建农村示范社区36个，创建综合减灾示范社区（村）30个。持续打造最具安全感省会城市。建立完善安全风险管控和隐患排查治理双重预防机制，坚决防止和有效遏制各类重特大事故发生。全面加大食品、药品、旅游、餐饮等行业监管力度，积极创建国家食品安全示范城市，切实规范市场秩序，维护消费者合法权益。继续做好国防教育及民兵预备役等工作。加强重大决策社会稳定风险评估，认真落实信访工作责任制，有效化解社会矛盾，确保社会和谐稳定。

切实加强政府自身建设

站在新的历史起点，肩负实现哈尔滨全面振兴的光荣使命，我们要以深入落实党的十八届六中全会精神为统领，进一步增强政治意识、大局意识、核心意识、看齐意识，努力建设人民满意政府。

（一）推进法治政府建设，打造全面振兴好环境。引领改革破障闯关、实现市场公平竞争，必须把法治政府建设摆在更加突出位置。牢固树立法治意识。积极推行政府常务会议前学法、法律顾问等制度，健全重大决策制定执行合法性审查、纠偏和责任追究机制，切实做到法定职责必须为、法无授权不可为，真正让法治思维和法治方式在哈尔滨落地生根，使尊崇法治、敬畏法律成为政府每个公务人员的思想和行动自觉。深入推进依法行政。依法接受人大监督，自觉接受政协的民主监督，主动接受社会和舆论监督，进一步完善政府信息公开目录，整合政府部门数据资源，增强政策执行透明度，让权力始终在阳光下运行。着力加强规则约束。大力倡导做诚信之人、办诚信之企、建诚信政府，强化完善市企业诉求受理中心职能，加大企业信用信息公开和违约惩戒力度，突出抓好政府违约问题专项整治，切实做到“言出必行”、“有诺必践”，力戒“说空话”、“乱承诺”、“不兑现”，让诚信成为引领哈尔滨发展的精神风尚。要始终保持行政问责高压态势，以零容忍的态度、猛药去疴的决心和壮士断腕的勇气，向那些奉行“潜规则”、造成“中梗阻”、损害哈尔滨全体人民整体利益的人和事亮剑，坚决纠正、从严惩处吃拿卡要、为官不为等破坏经济发展环境的行为，加快打造法治化的营商环境，使歪风邪气无处遁形，让我们这座城市始终充满公平正义的正能量！

（二）强化作风建设，提升政府执行力和公信力。要干事创业就要有好的作风。大力倡导“闯”的精神，大事难事看担当，在项目征收净地、产业引进培育、哈尔滨新区建设发展、国有企业改革、公共资产市场化运营等方面破旧立新、动真碰硬、担当作为，不断开拓工作新局面。大力倡导“实”的态度，坚持问题导向，知错就改，马上就办，努力在城市运行管理、公共服务供给、群众利益保障等方面干实事、求实效，以埋头苦干的真把式、雷厉风行的快把式、追求卓越的好把式，确保各项工作落到实处。大力倡导“廉”的风气，坚持把纪律和规矩挺在前面，以减权限权、创新监管等举措，缩小权力寻租空间，铲除滋生腐败土壤，切实让广大公务人员时刻心存敬畏、手握戒尺、干净干事。

（三）全面加强能力建设，更好地服务经济社会发展。推动哈尔滨全面振兴，需要一支素质过硬、敢于担当的公务员队伍。进一步强化学习增本领，自觉补充经济、科技、管理等领域的新理念新知识，及时了解基层和一线出现的新情况新问题，学深悟透用好国家系列政策，使政府决策更加符合实际、符合群众意愿，努力提高工作专业化水平。进一步严格考评促作为，强化工作绩效考核，坚决整肃庸政懒政怠政行为，健全正向激励和容错纠错机制，为干事者点赞、为担当者负责、为有为者撑腰，让明哲保身、投机钻营的没有市场，给恪尽职守、勤勉奉献、善作善成的改革创新者一展才华的广阔舞台。进一步增进协作提效率，牢固树立“全市一盘棋”思想，在谋划项目布局、落实管理责任、强化审批会办等方面加强统筹、有序配合，切实打破部门利益壁垒，坚决纠正推诿扯皮、办事拖沓等行为，努力形成汇智聚力谋发展、步调一致促落实的强大动力。

各位代表，振兴发展前途远大，重任千钧。正如总书记在新年贺词中所讲的那样，“天上不会掉馅饼，努力奋斗才能梦想成真”。让我们紧密团结在以习近平同志为核心的党中央周围，在省委、省政府和市委的正确领导下，与全市人民一道“撸起袖子加油干”，携手创造哈尔滨全面振兴新业绩，共同谱写人民美好生活新篇章！
